# End of life in Italy: ethical issues and medical-legal aspects of medically assisted suicide

**DOI:** 10.1007/s40592-025-00274-x

**Published:** 2025-11-13

**Authors:** Mariagrazia Marisei, Pierpaolo Di Lorenzo, Gaetano Di Donna, Verdiana De Caro, Fabio Policino, Claudia Casella

**Affiliations:** Department of Advanced Biomedical Sciences, University of Naples “Federico II”, Naples, Italy

**Keywords:** End of life, Court, Ethics, Palliative care, Medically assisted suicide

## Abstract

The debate on euthanasia and medically assisted suicide remains a complex issue, full of ethical, moral and legal implications, which has aroused strong emotions and conflicting opinions both in Italy and around the world. In Italy, the legal context surrounding euthanasia and medically assisted suicide is still characterized by an absence of specific rules regulating these practices. This has led to a situation in which the discussion on the end of life remains open and the practice of euthanasia remains illegal as the only interruption of life-sustaining treatments is allowed at present in accordance with the provisions of Law 219/17. Similarly, there is no specific rule concerning medically assisted suicide but only conditions identified by the case-law that make this practice not punishable in particular cases. In this delicate context, the role of the forensic physician is of paramount importance to ensure that these practices are carried out in a legal manner that respects the dignity of the patient. Starting from a description of the regulatory context of reference, a revision of news media cases that have been characterized by having added, from time to time, a piece to compose the current legal context, was conducted tracing analogies and similarities.

## Background

In recent years, the issues of the end of life and medically assisted suicide have taken on an increasingly important role in the scientific, political and philosophical debate (Jox et al. 2013; Svenaeus 2020; Sprung et al. 2018), not without contemplating some “extremisms” that see the individual supported by certain health treatments as “imprisoned in an artificial non-life (D’Aloia 2000).

In the Italian legal system, it is not yet possible to find a uniform regulatory discipline specifically aimed at the regulation of euthanasia.

In general, end-of-life practices represent conduct found in the Criminal Code in relation to Article 575 (homicide), Article 579 (homicide of the consenting party) and Article 580 of the Criminal Code (instigation or assistance to suicide).

Just as the Constitutional Charter does not explicitly affirm a right to life, neither does it propose an explicit or implicit duty to live. On the contrary, a reasoned interpretation of Articles 2, 13 and 32 of the Constitution seems to allow us to affirm that there is no coercible obligation to live (Flick and Dignity 2018).

Suicide itself, understood as the maximum expression of a “self-suppressive” will, is not provided for by law as a crime and is not punishable. The principle and the mandatory duty of social solidarity, proposed by Articles 2 and 4 of the Constitution, while justifying the social and ethical disapproval of the suicidal gesture as an escape from one’s social responsibilities, does not have the strength to legitimize a social or even juridical condemnation of that extreme gesture as a violation of an obligation of the author’s solidarity towards society and the family.

If medicalization is an increasingly present reality in all phases of an individual’s life, in responding to the needs of the community, constitutionally guaranteed goods must not be neglected. The law has always considered death as an instantaneous fact and has entered, first in distress and then in a short circuit, with the progressive change in the conditions of death.

The recent ruling by the Italian Constitutional Court that declared inadmissible the referendum question entitled “Partial repeal of art. 579 of the penal code (murder of the consenting party)” has sparked a heated debate, accompanied by reactions and controversies, which in some ways seem incongruous with the real scope of the measure and in others require in-depth reflection. The debate that ensued tended to focus on the general meaning to be attributed to the rejection of the question with respect to the broad and contrasting field of end-of-life management in conditions of illness or in any case of extreme suffering, of the related problems, of the claims and of the protection needs that can be traced back to it. The circumstance is also linked to the character and tone of the referendum campaign, in the context of which the idea that the referendum would have solved or at least contributed decisively to solving the problem of the so-called legalization of euthanasia has constantly been leveraged, in a way that is in many respects questionable. The referendum question called “Partial repeal of art. 579 of the Penal Code (homicide of the consenting person)” was declared inadmissible because, following the partial repeal, the minimum constitutionally necessary protection of human life would not have been preserved. In fact, as a result of the modifications proposed, the murder of the consenting person would have been rendered indiscriminately licit regardless of the reasons for consent, its forms of expression, the quality and characteristics of the perpetrator of the act and the ways in which death is caused (Cecchi et al. 2024; Marrone et al. 2022 Jul). If the referendum question had not been declared inadmissible by the Constitutional Court, the horizon of the discourse would have taken such a turn as to spread into a dangerous generalization (Cembriani 2021), in the wake of which the outline of the path traced by the Constitutional Court in the well-known judgment 242/19 (Constitutional and Court.^2019^. Decision No 242 of 22 November 2019 Constitutional appellate decision on cross-appeal.Official Gazette No48of 27 November 2019. 2019) would have been lost sight of. By ruling, within certain limits, the constitutional illegitimacy of art. 580 of the Criminal Code with regard to the case of assisted suicide, the issue has been linked to the dynamics of the care relationship and the discipline concerning terminality as provided for in art. 2 of Law 219/17 (Gazzetta Ufficialedelle Repubblica Italiana 2017).

Euthanasia is never mentioned in Italian criminal law (unlike the Spanish legal system) with the consequence that, in such a case, the Italian judges have so far qualified the offense by appealing to art. 575 (homicide), art. 579 (homicide of the consenting party) or, even more frequently, art. 580 of the Criminal Code (instigation or assistance to suicide) as it was declassified by the well-known sentence of the Constitutional Court in relation to the case of Dj Fabo, who was helped to die in a Swiss clinic by Marco Cappato (Constitutional Court, 22 November 2019, no. 242).

The Constitutional Court’s hope “that the matter be the subject of prompt and complete discipline by the legislator” was truly accepted in draft legislation no. 2553, approved by the Chamber of Deputies on March 10, 2022. With the subsequent fall of government, the draft legislation is stalled in the Senate committee. In general, it is considered that a person who is of age, capable of understanding and willing and of making free decisions, adequately informed and who has been previously involved in a palliative care pathway may apply for voluntary medically assisted death. It is specified that the applicant must be in the following concomitant conditions:

- been previously involved in a palliative care program in order to alleviate their state of suffering and have explicitly refused or voluntarily interrupted it;
- be suffering from a pathology attested, by the attending physician or specialist doctor treating him, as irreversible and with a poor prognosis;
- be affected by an irreversible clinical condition, and that such conditions cause physical and psychological suffering that the applicant finds absolutely intolerable;
- be kept alive by life-sustaining medical treatment, the interruption of which would result in the death of the patient.

The request must be current, informed, aware, free and explicit and comply with the procedures already provided for by the law on advance processing provisions (Law no. 219 of 2017).

In particular, the proposal provides that the request must be made in writing, in the form of a public deed or a notarized private deed.

If the person’s conditions do not allow compliance with these forms, the request may be expressed and documented by video recording or any other suitable device that allows him or her to communicate and unequivocally manifest his or her will in the presence of 2 witnesses. The request for voluntary death must be addressed to the general practitioner or to the doctor treating the patient, respecting the relationship of care and trust between patient and doctor. The physician will have the task of drawing up a detailed and documented report on the clinical, psychological, social and family conditions of the applicant and sending it to the territorially competent clinical evaluation committee. In the event of a favorable opinion, this must be sent to the health management of the Local Health Authority or hospital, which must guarantee death at the patient’s home where possible or at the facility. For health personnel, the possibility of conscientious objection is provided, without prejudice to the guarantee of completion of the procedures provided for by law in each region. The offences of assisting suicide and failure to render aid do not apply to the medical and administrative staff involved in the entire procedure. In relation to voluntary medically assisted death procedures carried out in compliance with the provisions of the law, the applicability to the doctor, health and administrative staff, as well as to anyone who has facilitated the patient in the execution of the procedure, of the crime of instigation or assistance to suicide, referred to in art. 580 and the crime of failure to provide assistance, pursuant to art. 593 c.p.

The series of human events and political cases that have enriched public opinion on the subject, bringing to light periodically conflicting considerations on what “life-support treatment” means and bringing new social awareness to the mixtures of opinions and positions, have helped to structure the path toward the definition of a law on the end-of-life.

In Italy, the first step proposed by the Constitutional Court in ruling No. 242/2019 and reaffirmed by ruling No. 135/2024 has been carried out only by law in the region of Tuscany. Although this law applies only within this region, it is still lacking a national regulation. Moreover, the State has filed a complaint with the Constitutional Court challenging the law for infringing on its legislative authority, and the matter is currently pending before the Court.

More specifically, Article 2 defines the requirements for access to medically assisted suicide, which are identified in the rulings of the Constitutional Court 242/2019 and 135/2024.

Article 3 defines the composition of the permanent multidisciplinary commission for verifying the existence of the requirements for access to medically assisted suicide and for verifying or defining the relevant implementation procedures. In particular, the commission is composed of a palliative care physician with expertise and experience in care, a psychiatrist, an anesthesiologist, a psychologist, a forensic physician, a nurse, and is supplemented on a case-by-case basis by a doctor specialized in the pathology affecting the person requesting access to medically assisted suicide. The members are identified, on a voluntary basis, from among the employees of the Local Health Authority.

The process takes a maximum of 50 days from the time the request is made until the procedure is completed.

## Materials and methods

The choice to present only some of the most well-known cases on the Italian scene stems from the desire to retrace the events that have determined the key rulings that have shaped the current cultural and regulatory framework on the subject of the end of life. The following will be a review of the news cases that have been decisive in shaping the current Italian regulatory scenario: the Englaro case, the Cappato case, the Trentini case and the Mario case, the Gloria case, the Sibilla case the Anna and the Serena case. In Italy, the procedure for medically assisted suicide is still being defined (Cecchi and Masotti 2020), as there is still no law that comprehensively regulates all aspects. At present, the sick person intending to carry out medically assisted suicide must contact a public structure of the National Health Service competent for the territory, directly or through the attending physician, in order to verify the requirements provided for by sentence no. 242/19 of the Constitutional Court (irreversible pathology, source of intolerable physical or psychological suffering, a person kept alive by means of life-sustaining treatment, a person capable of making free and informed decisions). In a first phase, the verification is entrusted to a special multidisciplinary commission appointed by the public structure. The Commission examines the request, accompanied by the supporting documentation, and discusses it with the interested party, ensuring the description of all possible alternatives, illustrating the palliative care pathway, including the use of continuous deep palliative sedation or adequate pain therapy and psychological support. The Commission is also responsible for initiating interviews, both with the person concerned and with family members and cohabitants. Once the evaluation phase has been completed, the Commission shall draw up a report expressing its opinion on the legality of the request. In the event of a positive opinion, the report is sent to the territorially competent Ethics Committee, which expresses its opinion on the case in question. It should be noted that the opinion of the Ethics Committee is mandatory but not binding, as it has advisory value. The favorable opinion of the Commission is necessary so that the public structure can provide the patient with the necessary medication and equipment to carry out, at any time he wishes, the procedure of medically assisted suicide.

### The Englaro case

The Englaro case (Moratti 2010 Jul; Solarino et al. 2011; Luchetti 2010; Associazione Luca Coscioni 2017) not only represented a serious human event capable of inducing a profound reflection on values but also constituted a field of confrontation between law, politics and the public health service. On 18 November 1992, at the age of 20, the young Eluana was the victim of a serious road accident with a consequent vegetative state until 9 February 2009, the date of implementation of the decree of the ordinary judge that authorized the interruption of artificial nutrition and hydration (Crepaldi 2022). The first “juridical stage” of the civil path is the declaration of interdiction of Eluana by the Court of Lecco in 1996 with the appointment as guardian of her father Mr. Beppino Englaro and the immediate start, by the latter, of the judicial process to obtain from the civil magistracy the authorization to interrupt the vital medical treatment. This authorization came with a decree of the Court of Appeal of Milan in July 2008, following a referral by the Court of Cassation (Court et al. 2007) (judgment No. 217548/2007). It is possible to deactivate the health device of artificial nutrition and hydration of patients in a permanent vegetative state when two conditions exist: a requirement of severity - the vegetative state is not subject to any recovery of consciousness (an aspect that distinguishes the Englaro case from the Welbi and Dj Fabo case) - and a temporal requirement - the vegetative state must have lasted for a long time with consequent total inability to relate to the outside world.

With its ruling, the Supreme Court underlines an important principle of law that sees the legal guardian entrusted with the “care” of the incapacitated person pursuant to art. 357 and 424 of the Italian Civil Code, entrusting him with the task of deciding in the absolute interest of the incapacitated person, expressing a will attributable to the latter and deducible from his previous life (Tripodina 2008). The decree of the Court of Appeal was subsequently appealed before the Court of Cassation, which declared it inadmissible due to lack of legitimacy of the Public Prosecutor. Alongside the civil and political issues, the administrative proceedings for the failure to comply with the decisions taken in civil court consisted in the denial of hospitalization in various Lombardy regional health facilities in order to implement the sentence of the civil judge who had authorized the suspension of artificial feeding treatment. In the course of the affair, the Region went so far as to state that health personnel could not proceed with the suspension of hydration and artificial feeding since, otherwise, they would have failed in their professional obligations and fulfillments.

Since he had no chance of being guaranteed enforcement in civil proceedings, the legal guardian brought an action before the Administrative Court. Eluana died on 9 February 2009 at the private care facility “La Quiete” in Udine, the only one that made available to her the implementation of the civil court. The administrative appeal judge held that the Region had refused the first hospitalization for the interruption of forced feeding on the basis of a vision of care that “subjectivizes” the provisions of art. 32 of the Italian Constitution, in the sense of requiring a necessary benefit for the patient, setting aside the consideration of therapeutic will and self-determination, which is the highest expression of personality. Not only that, but in accepting the Englaro family’s claim for compensation, the Lombardy Regional Administrative Court recognized the existence of fraud, having “deliberately and knowingly” left the civil judgment unfulfilled.

In the opinion of the Regional Administrative Court, the incriminating factor of the “reason of conscience” applies only to “individuals”, “while the conscience of the institutions is constituted by the laws that regulate them”. Informed consent establishes a doctor-patient relationship that is functional to the definition, in a dynamic, flexible and personalized way, of the care needs and therapies to be implemented. The resulting decision must also include, in the extreme, non-care, rejection and abstention; The health administration must interrupt treatment in cases where the patient or, in the case of incapacity, the legal guardian, has unequivocally expressed the desire to renounce it, considering this choice more suited to the personality, the way of living and understanding life and the idea of a dignified existence that the patient has manifested or can manifest expressly.

### The Dj Fabo case

With regard to assisted suicide, the Dj Fabo case (Cupelli 2019; Palermo Fabris 2020; Montanari 2019; Delbon et al. 2021; Stampanoni Bassi 2017) has led to two constitutional pronouncements, arising from the regulatory vacuum that has forced the Constitutional Court to replace or, better, constitute itself as a Legislator given the social relevance of the pending problem. It all stems from Marco Cappato’s self-denunciation to the Carabinieri of Milan to which Cappato went, representing that he had gone to Switzerland with Fabiano Antoniani, known as Dj Fabo, to help him implement his intention of assisted suicide.

After a series of hearings and examination of key witnesses in the case, the Court of Assizes of Milan issued an order in which it raised the constitutional legitimacy of Article 580 of the Criminal Code (“assisted suicide”). By order no. 207 of 2018, the Constitutional Court pointed out that the rule applicable in the criminal proceedings against Marco Cappato would have been declared constitutionally illegitimate, if the Parliament had not taken steps to innovate the regulatory discipline dictated on the subject of assisted suicide. The constitutional process went on until September 2019, the date on which, with sentence no. 242 of 2019, Article 580 of the Criminal Code was declared partially unconstitutional. In its ruling, the Court, without modifying the incrimination of the conduct of assisting suicide, identifies an area of constitutional non-compliance of the incriminating conduct in the presence of four subjective conditions that can be found in the patient. The pronunciation, therefore, defines four conditions for the patient that decriminalize the doctor’s conduct with respect to the type of crime referred to in Article 580 of the Criminal Code, namely: patient suffering from irreversible pathology; that it is a source of physical or psychological suffering that is considered unbearable; the patient is kept alive by life-sustaining treatment; the patient should be able to make free and informed decisions. The requirement mentioned above as third in the order of the list will be returned to later, when the case involving Davide Trentini, managed on the merits by the Court of Assizes of Massa, will be illustrated. This interpretation, innovative and partly corrective of the previous ruling, has been followed in Europe by both the German Constitutional Court and Spain, where in 2021 the “Ley Organica de regulación de la euthanasia” was approved (Law and 3, 2021, of 24 March, on the regulation of euthanasia.BOEno. 72, of March2021, 25, pp. 34037–49.2021). The four conditions of the patient identified by the Court as discriminating from the conduct of medically assisted suicide are analyzed in detail. The first requirement, that of “irreversible pathology” has generic connotations, being able to range from progressive neurological conditions to post-traumatic ones to oncological pathologies. Secondly, the pathology found must induce an “intolerable psychic and/or physical suffering” of an exclusively subjective nature, as it is calibrated on the personal perception and tolerance of suffering. The third requirement that is offered as a decriminalizing factor for the conduct of assisting suicide is that the patient be kept alive by “life-sustaining treatments”, i.e. medical devices of forced nutrition and hydration, mechanical ventilation or continuous renal replacement therapy.

The requirement of “life support”, as defined by the Court, has also been criticized by authoritative voices. Such a prerequisite would create “*unreasonable and unconstitutional discrimination [...] between those who are kept alive artificially and those who, although suffering from this pathology, even the most serious and suffering greatly, are not or are not yet so”* (D’Avack 2020; Casonato 2020). The broad interpretation of “vital treatment” provides for the extension to any health treatment capable of guaranteeing the patient a longer life expectancy than that which would be expected if no health intervention were practiced.

For the legitimacy of the choice, the “patient must be able to make free and informed decisions”. The reference to the “capacity to act” is linked to the provisions of paragraph 5 of art. 1 of Law No. 219 for refusal of therapies. The “absence of the legislative power” (Masoni 2021) has not prevented the Constitutional Court from already identifying “in the present regulatory system a precise point of reference, which can be used for the purposes considered”, namely articles 1 and 2 of Law 219 of 2017. Assisted suicide is equated with the refusal of therapeutic treatment. As for the procedure, with the affirmation of the legitimacy of assisted suicide and the consequent non-criminal liability of the doctor who participated in the conduct of suicidal aid, the Court referred the verification of the legitimizing conditions of access to the “service” (which will be discussed more fully below) and discriminating with respect to the conduct to the National Health Service, in order to avoid “abuses to the detriment of vulnerable people”, subject to the opinion of the Territorial Ethics Committee. However, the possibility remains for the doctor, in the face of the request of the patient capable and able to self-determine, to “choose whether or not to lend himself to fulfilling the patient’s request”. Contrary to the provisions of the sixth paragraph of art. 1 of Law 219/17 (in the event of refusal of treatment, the doctor is left with no margin of discretion, as he is bound to carry out this manifestation of will) in the case of assisted suicide the choice of the doctor is left to his free conscience. The work of the Constitutional Court has made it possible not to submit to the inertia of the Legislator in the face of the urgency of dramatic cases that for too long have not been accepted in a container of secularism such as to allow the maximum expression of self-determination (Zagrebelsky 2010).

The great upheaval in the field of ethics committees has led to a sort of “disarray” in the aftermath of the Constitutional Court’s ruling, with the widening of the range of competences specifically attributable to the specific collegiate body. The opinion of the ethics committee is mandatory but not binding. However, legislative action would be necessary to adapt the composition and powers of ethics committees. Combining the two ordinances, the interlocutory one no. 207/18 and no. 242/19, there has been a complete downsizing of the roles and functions of those who intervene in the path of a terminal patient and a substantial change in the criminal liability profile of the health worker who assists the suicide, which results in non-criminal conduct. The innovative faculty, i.e. the right to request access to assisted suicide, is linked to the fundamental rights enshrined in Law 219/17: the right to refuse treatment (art. 1, paragraph 5); the right to request the application of analgesic therapy, to benefit from palliative medicine and deep palliative sedation (art. 2, l. 219) as well as the right to plan one’s own health future (art. 4 and 5 of l. 219). The guarantee of protection “in order to avoid abuses to the detriment of vulnerable people” is given by the intervention of public structures of the National Health Service.

Well before the Court’s final ruling, the Italian Bioethics Committee (CNB, Italian acronym for “Comitato Nazionale per la Bioetica”), in an opinion dated 18 July 2019 (Committee 2019), had illustrated that “*there are various positions, but basically they can be traced back to two different perspectives that turn out to be in contrast with each other*”.

The first affirms that “*any involvement in practices aimed at causing death (assisted suicide or euthanasia) would entail a profound change (or even a distortion) of the figure of the doctor and his role in health facilities and of the health facilities themselves. In fact, instead of being aimed at helping in dying, that is, accompanying in dying through palliative care and pain therapy, these would be directed to help in dying through collaboration in (or the execution of) acts that directly cause death. Excluding assisted suicide allows the doctor to preserve the ethical-deontological significance of his profession and the patient to maintain more firm and solid trust in his doctor*”. The second, more recent and less widespread position states that helping to die “*may be part of the professional duties of the doctor and health care personnel*” with a view to not causing any harm to the patient. Quoting the exact words that appear in the same pronunciation: “*Today not only have the conditions of dying changed profoundly compared to the past, also because people want to assert self-determination over their own life and death. In some cases, the process of dying is prolonged by medical interventions that involve suffering and anguish in people, so that not only palliative care and care planning programs are required, but there is also an explicit request for help in dying to overcome an inevitable situation of pain. In these cases, the doctor’s willingness to comply with the request to die arises from the primum non nocere, that is, from the duty that is imposed not to cause harm and to reduce pain*”*.* Therefore, there is a sort of commitment of the doctor who no longer and not only treats the sick by intervening on the pathology that afflicts him, but also the right to alleviate the patient’s suffering, to the point of providing him with help for a pitiful and dignified death. As an immediate effect of the Constitutional Court pronouncement, an adaptation of the code of ethics is required in the light of the redefinition of the doctor’s task from a modern perspective, more in keeping with the values that health in the third millennium brings into play and which are those outlined in the debut norm of Law 219/17. Article 3 of the Code of Medical Ethics is entitled “General Duties and Competencies of the Doctor” and provides that “the duties of the doctor are the protection of life, psycho-physical health, the treatment of pain and the relief of suffering, with respect for the freedom and dignity of the person, without discrimination of any kind [...]”. In art. Article 17 explicitly prohibits the physician from “encouraging acts aimed at promoting death”. At the meeting of 6 February 2020, the deontological rule was adapted to the Cappato ruling. The deontological dictate provides that: *“the free choice of the doctor to facilitate, on the basis of the principle of self-determination of the individual, the intention to commit suicide autonomously and freely formed by a person kept alive by life-sustaining treatments suffering from an irreversible pathology, a source of intolerable physical or psychological suffering, who is fully capable of making free and informed decisions [...] it must always be assessed on a case-by-case basis and entails, if all the elements indicated above exist, the non-punishability of the doctor from a disciplinary point of view”.* Apart from the reflection on deontology which defines that there is no obligation on the part of the doctor to comply with the patient’s wishes, there is an obligation on the National Health Service, with a view to guaranteeing everyone the right to a dignified death, in accordance with the principle of effectiveness (Gorgoni 2020).

### The Trentini case

Thanks to the ruling of the Constitutional Court, it was easy to reach the resolution of another important case, that of Davide Trentini (Corte d'Assise di Massa 2020; End of life 2021), for which Marco Cappato and Mina Welby were accused of the crime referred to in art. 580 of the Criminal Code, having materially helped Trentini in the execution of the suicidal intention. The acquittal of the defendants took place in full because “the fact does not exist”. From the ruling of the Court of Assizes it can be deduced that the requirement of dependence on life-sustaining treatments - supplementary to the depenalization introduced by the Constitutional Court with ruling no. 242 of 2019 - is also found when the patient does not depend on a machine but on health treatments carried out with pharmacological therapies or with the absence of medical or paramedical personnel, without which a process of weakening of the organism would be triggered, which would inevitably lead to its death (Cappelli 2021). The case that led to the decision of the Court of Assizes relates to the request for medically assisted suicide of Davide Trentini, who suffered from multiple sclerosis and died in Switzerland on April 13, 2017. In the long course of the disease, which had arisen 25 years earlier, Trentini had experienced a rapid and inexorable deterioration in recent years with the need to resort to invasive medical procedures in the face of the impossibility of alleviating suffering with pain therapy. The Court of Assizes of Massa held that the fact does not exist with reference to the conduct of reinforcement of the suicidal intention and that the fact does not constitute a crime with regard to the conduct of facilitating the execution of suicide. With regard to the first charge, the Court excluded the conduct of reinforcing the suicidal intent on the basis of the witness evidence acquired and, on the second charge, that there were additional elements of crime, or the doubt as to their existence as introduced by the Constitutional Court by ruling no. 242/2019. The focal point of the Tuscan Court’s ruling, the one that makes the clinical and judicial case of Trentini particularly interesting for the discourse that we intend to build with this paper, concerns the requirement of dependence on life-sustaining treatments that finds a new interpretation. This requirement does not result in dependence on a machine, but in pharmaceutical, medical and paramedical health treatments, without which the patient would inevitably move towards a process of weakening of organic functions, the outcome of which—not necessarily rapid—is death. The Court of Assizes had two alternatives: to raise the question of constitutional legitimacy or, alternatively, to opt for a broad interpretation of the requirement in question. The broad interpretation of the requirement is based on a twofold consideration on the part of the courts on the merits. Addiction to life-sustaining treatments does not necessarily mean addiction to a machine. The second consideration relates to a legal “artifice”. The Court of Assizes used the analogy to acquit the defendants. In criminal matters, the prohibition of analogy applies only to the interpretation of criminal provisions unfavorable to the offender. In this sense the application by analogy of the provisions laying down discriminatory norms is considered permitted. Summarizing a rule of criminal law on analogy, it is concluded that the requirement of dependence on life-sustaining treatment has also been considered to be applicable by analogy to similar situations. With the broadening of the notion of life-sustaining treatment and the use of the analogy, the judgment made a significant correction to one of the profiles of the constitutional pronouncement that the doctrine had most criticized. The application of the principle of equality on which the Constitutional Court’s argument is based leads to the unreasonable view pursuant to Article 3 of the Constitution, of not only the unequal treatment between those who, dependent on life-sustaining treatment, refuse such treatment and those who, in the same condition of dependence, decide to be helped by others in killing themselves; as well as the unequal treatment that would be created between those who, dependent on a machine, can resort to medically assisted suicide and those who, in the same pathological condition, cannot do so because they are subject to medical treatments of different types but still life-sustaining. In all cases of life-sustaining treatment, the patient – provided that all other requirements are also met – exercises his or her right to therapeutic self-determination in a space within which the application of art. 580 of the Criminal Code is to be considered constitutionally unreasonable.

### The Mario case

This is the first request (Associazione Luca Coscioni 2021; Ridolfi 2022; Andreani Teresa 2022) for access to “assisted dying” in the aftermath of the Constitutional Court’s ruling on the Dj Fabo case. Mario is a fictional name that accompanied the entire clinical and chronicle affair, which ended in June 2022 with the death of the later revealed Federico Carboni. He was a man born in 1978 and living in a small village in the Marche region who, following a serious accident on the road, had become quadriplegic. In September 2020, having matured the intention of accessing medically assisted suicide after the ruling of the Constitutional Court, he addressed a request to the Local Health Authority to which he belonged for verification of the existence of the conditions indicated by the Constitutional Court to access this service. Local Health Authority even denied Mario the procedures for verifying the existence of the access requirements and it is from this moment that the legal battle began, which ended, as anticipated, in June 2022. The distinctive feature of the case is that it starts in the civil field and not in the criminal one. Mario resorted to an emergency procedure pursuant to Article 700 of the Code of Civil Procedure, but was ruled against by the Court of Ancona, which came in March 2021. The Court, while acknowledging that the patient has the requirements that have been provided for by the Constitutional Court in judgment 242/19, states that *“there are no [...] reasons to believe that, by identifying the cases in which assisting suicide can now be considered lawful, the Court has also founded the patient’s right, where such cases occur, to obtain the cooperation of health professionals in implementing his decision to end his or her life; nor can it be considered that the recognition of the right invoked is a direct consequence of the identification of the new hypothesis of non-punishability, taking into account the multifunctional nature of the criminants not always instrumental to the exercise of a right”.*

Following a complaint, the same Court, this time in collegial composition, overturned the previous decision, requiring the Marche Regional Health Authority to verify whether the conditions for access to assisted suicide were respected in Mario’s case and to ascertain whether the methods and drug chosen were suitable to guarantee him the fastest and most dignified death possible. A month after the ruling of the Court of Ancona in 2021, Mario was forced to warn the Marche Regional Health Authority. Finally, in his intentions, in September 2021 Mario was offered a schedule of appointments aimed at verifying the conditions for carrying out the preparatory checks required by the Marche Region Ethics Committee to issue its opinion. In October 2021, the patient was notified of the sending of the collegial report drawn up by the interdisciplinary team dedicated to the Marche Region Ethics Committee. After a further warning due to the delays of the operating procedure, on 23 November 2021 Mario received the opinion of the Ethics Committee which found the presence of the 4 conditions established by the Constitutional Court, noting the impossibility of being able to express himself on the lethal drug. However, neither the Company nor the Ethics Committee had expressed an opinion on a step not only ordered by the Court but also prescribed by the judgment of the Constitutional Court, inducing Mario to submit yet another warning. In fact, the Ethics Committee and the Marche Regional Health Authority are denounced for the crime of torture. On 9 February 2022, the report of the multidisciplinary technical group indicated via mail method and drug chosen. The commission was composed of two directors of complex operating units (Anaesthesia-Resuscitation and Forensic Medicine), two directors of simple departmental operating units (palliative care and pharmacy), a full professor of Pharmacology and an Asur manager. The final decision stated that: “Thiopentone sodium appears suitable to ensure a rapid and painless death at a dosage of not less than 3–5 grams for an adult person weighing 70 kg. The mode of administration is that of self-administration by intravenous infusion”. With the validation of the drug and the methods of administration, a precedent was created in the implementation of the Constitutional Court’s decision. In the absence of a law that regulates obligations and responsibilities in the exercise of a right, that of self-determination, it remains the responsibility of the citizen to find the drug, have it prescribed by a private doctor and procure the (expensive) equipment necessary for self-administration, effectively excluding this service from the list of those that can be implemented within the National Health Service. On June 16, 2022, Mario’s destiny was fulfilled.

### Gloria case

The second Italian (ANSA 2023) to resort to medically assisted suicide was Gloria (fantasy name), a 78-year-old cancer patient from Veneto. The request was forwarded to the competent Local Health Authority on 23 November 2022. After carrying out the appropriate checks, the positive report of the multidisciplinary group was forwarded to Gloria on 30 March 2023. In particular, in this case, anticancer drugs were equated with real life-sustaining treatments. However, similarly to what happened in the Mario case, the Local Health Authority did not immediately identify the lethal drug and the methods of self-administration. Gloria’s defense lawyers, therefore, invited the Local Health Authority to provide the missing information. On 7 June 2023, it was confirmed that the drug and the necessary equipment would be provided by the Regional Health Service, also communicating that there were no medical personnel available to provide their assistance. In the meantime, there were further deteriorations in Gloria’s state of health who, on June 22, 2023, sent a new urgent request for the supply of the drug and the necessary equipment, communicating that she would be assisted by a doctor of her trust. On 17 July 2023, a new visit was ordered to ensure that Gloria was still capable of understanding and wanting and that her will had not changed, which was indeed noted. As all the requirements provided for by the relevant case law were met, on 23 July 2023 Gloria, assisted by her trusted doctor, self-administered the lethal drug and died at her home. Three important steps forward were achieved compared with the previous Mario case. Firstly, there was no need for legal proceedings, which made the process more streamlined. Secondly, the chemotherapy treatment to which Gloria was subjected was regarded in all respects as life-sustaining treatment, falling within the requirements identified by the Cappato judgment. Finally, the drug and the equipment for self-administration were provided free of charge by the competent Local Health Authority.

### Sibilla case

She was a cancer patient (ANSA 2023) who was accompanied by her son to Switzerland to practice assisted suicide after the Local Health Authority in Italy had refused the access to this procedure because of the patient’s non-dependence on life-sustaining treatments. Although the local ethics’ committee had recognized the presence of the four conditions required by the Constitutional Court judgement, the Local Health Authority opposed this decision since they affirmed that the current conditions were not consistent with intolerable physical suffering.

### Anna case

On November 28, Anna (Associazione Luca Coscioni 2023; Anna 2024), a 55-year-old woman suffering from progressive multiple sclerosis who had applied for assisted suicide, died. She was the first Italian citizen to obtain assisted suicide in Italy with the full assistance of the National Health Service, which covered the expenses for the drug to obtain the necessary equipment to complete the procedure.

The assisted suicide took place under the supervision of a doctor identified by the health authority who, on a voluntary basis, assisted Anna without intervening directly in the administration of the drug, an action that remained “the exclusive responsibility” of the patient. “Anna” (not her real name, chosen to protect her privacy) was a woman of about 55 years old, suffering from rapidly evolving progressive multiple sclerosis, making her less and less independent and more dependent on the assistance of third parties. Although the epilogue of the affair went in the direction of the patient’s will, it was necessary to make an urgent appeal before the Court and a complaint to the Public Prosecutor’s Office to solicit the intervention of the structures in charge in accordance with the provisions of the Constitutional Court’s ruling.

### Serena case

Serena (fantasy name)was a woman who had been suffering from progressive multiple sclerosis for about 30 years. Her condition, which gradually paralyzed her, forced her to live in a situation of total dependence, requiring continuous assistance from her caregivers. In May 2024, Serena applied to verify the conditions in order to access medically assisted suicide. The Local Health Authority, after conducting home visits and obtaining the opinion of the competent local ethics committee, verified that all the requirements indicated by the Constitutional Court had been met. However, they did not specify the lethal drug and the equipment for its self-administration, pointing out that a collegial assessment with the hospital pharmacist would be necessary.

Serena's lawyers highlighted that the health authority is in charge of providing the necessary assistance to access medically assisted suicide, including the prescription and supply of the lethal drug, the equipment for self-administration, and the healthcare personnel who, on a voluntary basis, provide their voluntary assistance during the procedure.

In December 2024, in the absence of a response from the health authority, Serena's lawyers sent a reminder requesting an urgent response, given the continuing deterioration of her condition. After evaluating the report with the pharmacological protocol from Serena's trusted physician, the Local Health Authority confirmed that it would provide the lethal drug and the equipment for self-administration, but that it had been unable to find healthcare personnel willing to provide assistance on a voluntary basis. In January 2025 Serena, assisted by her trusted physician, self-administered the lethal drug in her own home.

### Discussion (and the role of the forensic physician at the end of life)

Anna is the first sick person who, following a Precautionary Order issued by the Court, met all the requirements provided for by the Cappato sentence of the Constitutional Court and was able to access voluntary death entirely at the expense of the National Health Service. “Mario” was the first person to resort to assisted suicide in Italy: he had to personally take charge of the drug, find a doctor to prescribe it and a machine (the drug and the machine had been purchased thanks to a fundraiser); Gloria, the first person in Italy to die through assisted suicide without having to go through a court, had received the drug from the National Health Service; in Anna’s case, the Health Service provided drugs, equipment and a doctor Table 1

**Table 1:** Synoptic table of the most relevant Italian cases

Case	Reference legislation	Pathology	Geographical place of death	Structure in which it was made	Modality	Intervention of the court
Englaro	Criminal Code	Post-traumatic permanent vegetative state	Italy	Public Facility	Suspension of life-sustaining treatment	Yes
Dj Fabo	Criminal Code	Post-traumatic quadriplegia	Switzerland	Private Facility	Assisted suicide	Yes, after the affair for self-denunciation
Trentin	Criminal Code	Multiple sclerosis	Switzerland	Private Facility	Assisted suicide	Yes, after the affair
Mario	Judgment 242/19	Post-traumatic quadriplegia	Italy	home	Medication provided by NHS, instrumentation and patient care	Yes, first, for failure to initiate the required procedure.
Gloria	Judgment 242/19	tumour	Italy	Home	Physician-assisted suicide	No
Sibilla	Judgment 242/19	tumour	Switzerland	Private Facility	Assisted suicide	Yes, after the affair for self-denunciation
Anna	Judgment 242/19	Multiple sclerosis	Italy	Home	Medically assisted suicide, at the total expense of the NHS with identification of the doctor on a voluntary basis	Yes, in the course of the affair to urge the implementation of the procedures
Serena	Judgment 242/19 and Judgment 135/24	Multiple sclerosis	Italy	Home	Physician-assisted suicide (no NHS). Drug and instrumentation provided (NHS)	No

Euthanasia and medically assisted suicide are controversial practices that raise complex ethical and legal issues (Claudia et al. 2024). Euthanasia is an illegal practice in Italy, as only the interruption of life-sustaining treatments is allowed at present in accordance with the provisions of Law 219/17. Similarly, there is no specific rule concerning medically assisted suicide but only conditions identified by the case-law that make this practice not punishable in particular cases. In this delicate context, the role of the forensic physician is of paramount importance to ensure that these practices are carried out in a legal manner that respects the dignity of the patient. In the previous paragraphs, the issue of access to the practice of medically assisted suicide of the patient who wants to resort to it has been discussed. This practice involves the intervention of two institutions, namely the Local Health Authority of the National Health Service and the reference Ethics Committee. In the first case, the forensic physician becomes part of the multidisciplinary Commission, assuming the role of coordinator. In this regard, it should be pointed out that nothing is provided for a priori in the current legislation on the exact composition of the aforementioned Technical Commission (as it is instead stated only in Tuscany) and, therefore, its convocation is entrusted to the discretion of the Territorial Companies. In this context, the role of the forensic physician is carried out in verifying the requirements of the “Cappato” Judgment, in order to ensure that the entire procedure is carried out appropriately. This includes reviewing medical documentation and informed consents, verifying the patient’s will, in order to make sure that the decision to resort to medically assisted suicide is free, informed and conscious. The opinion of the Local Health Authority Commission, when favorable, must be submitted to the scrutiny of the territorially competent Ethics Committee, albeit with an advisory value only. The Italian Bioethics Committee, through a press release in March 2023, established the composition of this Committee but, paradoxically, did not expressly provide for the figure of the forensic physician. Although the members of this commission must include a “bioethicist” and an “expert in law”, the SIMLA (Italian Society of Legal Medicine and Insurance) has raised doubts about the identification of these members and suggested that the figure of the forensic physician should be expressly provided for among them.

In fact, the importance of the forensic expert has been well recognized by the Tuscan law.

Another function that the forensic physician could be called upon to perform in the context of the end of life is judicial advice, both for the Office and for the party. Precisely by virtue of the lack of legal regulations, jurisprudence will increasingly be called upon to resolve disputes that may arise between the requesting citizen and the territorial authorities competent to issue the technical opinion. In the case of the Mario case, which has already been discussed. Similar legal disputes may also arise with regard to Law 219/2017, where questions relating to the lawfulness of consent may be raised. Also in this case, the forensic physician may be called upon to express his technical opinion on the merits. A particular task attributable to the forensic physician, again in the judicial field, could be, in patients who died as a result of these practices, the ascertainment of the reality of the death, if it were suspected that it could be the consequence of other causes potentially attributable to crimes. A further profile of study and reflection is constituted by the conscience clause that could be invoked at the request that a doctor employed by the NHS be called upon to assist during the preliminary procedures for the self-administration of the drug for suicidal purposes. Even in the face of this circumstance, medico-legal skills appear to be of crucial importance in the context of Local Health Authorities.

## Conclusions

The growing attention to end-of-life issues, both from civil society and institutions, suggests that the country may be on the way to addressing these problems in a more in-depth and comprehensive way. The desirable action of the Legislator in proposing a legal regulation in the form of a “framework law” on the matter of the end of life will certainly require the establishment of a technical table where the primary role should be attributed to the forensic medical component.

Without a primary-ranking law, Tuscany’s regional law is undoubtedly a good place to start, but it may impede the National Health Service’s need of universality. Furthermore, in terms of end-of-life practices, a national legal standard would maintain the generality and abstractness requirements that apply to all citizens and those receiving assistance from the National Health Service without worrying about causing care disparities that would disadvantage citizens in other parts of the country and contribute to the already well-known phenomenon of “health tourism”.

## Data Availability

No datasets were generated or analysed during the current study.

## References

[CR3] Andreani Teresa, 2022. Azienda Sanitaria Unica Regione Marche : report of Multidisciplinary Technical Group on modality, method and lethal drug for assisted suicide procedure in Mario case Accessed 20 August 2024 https://www.biodiritto.org/Biolaw-pedia/Docs/Azienda-Sanitaria-Unica-Regione-Marche-relazione-del-Gruppo-Tecnico-Multidisciplinare-su-modalita-metodica-e-farmaco-letale-per-procedura-di-suicidio-assistito-nel-caso-Mario.

[CR1] Anna. 2024. Anna died; she had requested assisted suicide. For the first time, the drug provided by the National Health Service Accessed 21 August 2024 https://www.rainews.it/articoli/2023/12/la-prima-in-italia-muore-a-trieste-la-donna-che-aveva-chiesto-il-suicidio-assistito-cf722571-3448-493a-ad61-3c73b697df21.html.

[CR2] ANSA (National Associated Press Agency), 2023a. Assisted suicide, actress Sibilla Barbieri dies in Switzerland Accessed 21 August 2024 https://www.ansa.it/sito/notizie/cronaca/2023/11/06/suicidio-assistito-lattrice-sibilla-barbieri-muore-in-svizzera_b8937d75-765d-45a6-b42b-0e726877acc5.html.

[CR17] ANSA (National Associated Press Agency), 2023b. Gloria died, second case of assisted suicide in Veneto region Accessed 21 August 2024 https://www.ansa.it/veneto/notizie/2023/07/24/e-morta-gloria-secondo-caso-di-suicidio-assistito-2_9e7e32cd-502c-452a-87d4-52e26f84da3f.html.

[CR39] Associazione Luca Coscioni, 2017. The court case of Eluana Englaro Accessed 21 August 2024. https://www.associazionelucacoscioni.it/caso-giudiziario-eluana-englaro.

[CR38] Associazione Luca Coscioni, 2021. The case of "Mario" who seeks assisted death in Italy and takes Local Health Authority to court point by point Health Authority-punto-per-punto. Accessed 19 August 2024 https://www.associazionelucacoscioni.it/il-caso-di-mario-che-chiede-la-morte-assistita-in-italia-e-porta-in-tribunale-la-asl-punto-per-punto

[CR37] Associazione Luca Coscioni, 2023. The case of Anna (2023). Accessed 16 August 2024 https://www.associazionelucacoscioni.it/il-caso-di-anna.

[CR4] Cappelli , V. 2021. The notion of “life-sustaining treatment” after Constitutional Court ruling 242/2019: the Trentini case. https://www Accessed 22 August 2024.

[CR5] Casonato, C. 2020. The Constitutional Court between just attention and excesses of caution, in Nuova giur.civ.comm, 2, 418.

[CR6] Cecchi, R., and V. Masotti. 2020. Medically assisted suicide: what future prospects for the physician? *Italian Journal of Forensic Medicine* 1 (2020): 414–419.

[CR7] Cecchi, R., M. Sassani, F.S. Romolo, A. Rovito, M. Montisci, V. Masotti, et al. 2024. Medically assisted suicide in Italy: Recent legal developments on a controversial topic. *Medicine, Science and the Law* 64 (1): 77–81. 37306159 10.1177/00258024231182373

[CR8] Cembriani, F. 2021. Euthanasia on the test of the popular repeal referendum and its dangerous pitfalls. *Italian Journal of Forensic Medicine* n 4/21.

[CR9] Claudia, C., C. Emanuele, M. Mariagrazia, D. Donna. Gaetano, D. Lorenzo Pierpaolo, and N. Massimo. 2024. Exploring health professionals’ knowledge of end of life in Italy. *Legal Medicine* 71: 102542. 39447468 10.1016/j.legalmed.2024.102542

[CR24] Corte d'Assise di Massa, 2020. Judgment n. 1 of 27/07/2020 of the Court of Assizes of Massa available at Accessed 21 August 2024 https://www.sistemapenale.it/pdf_contenuti/1600007359_corte-assise-massa-trentini-assoluzione-cappato-welby-suicidio-assistito.pdf

[CR10] Crepaldi, G. 2022. The Englaro case: the civil and administrative judgments and the denouement before the Court of Auditors. in Civil Liability and Welfare - n. 1-2-2022.

[CR11] Cupelli, C. 2019. The Cappato case: self-determination and dignity in dying. In *Il caso Cappato Riflessioni a margine dell’ordinanza della Corte costituzionale n, *ed*. *C.C. Marini F.S, 207 del 2018 75-90. ESI.

[CR12] D’Aloia A. 2000. Law and rights in the face of death. Hypotheses and questions around the normative regulation of euthanasia behaviors. *In Chieffi L. Bioethics and Human Rights.*

[CR13] D’Avack L. 2020. Informed consent and end-of-life choices, Torino

[CR14] Delbon, P., F. Maghin, and A. ContI. 2021. Medically assisted suicide in Italy: the recent judgment of the Constitutional Court. *LA CLINICA TERAPEUTICA.* 3:193–6. 10.7417/CT.2021.2312 33956035

[CR15] End of life: Genoa Court of Appeal’s acquittal of Mina Welby and Marco Cappato in death of Davide Trentini (2021) https://www.giurisprudenzapenale.com/2021/07/19/fine-vita-la-sentenza-di-assoluzione-della-corte-di-assise-di-appello-di-genova-nei-confronti-di-mina-welby-e-marco-cappato-per-la-morte-di-davide-trentini/. Accessed 20 August 2024

[CR33] Fabio Ridolfi died after initiating deep sedation (2022) https://www.ansa.it/marche/notizie/2022/06/13/e-morto-fabio-ridolfi-dopo-aver-avviato-la-sedazione-profonda_f7629804-7daa-4e86-9488-6cb3e89c9fad.html. Accessed 14 August 2024

[CR16] Flick, G.M. 2018. Dignity in living and dignity in dying: a (cautious) step forward. In *The right on life. Living will, self-determination and the dignity of the person*, ed. V. Verduci, Pisa, 17 ff.

[CR25] Gazzetta Ufficiale delle Repubblica Italiana, 2018. Law n 219/2017. Provisions for informed consent and advance treatment directives https://www.gazzettaufficiale.it/eli/id/2018/1/16/18G00006/sg

[CR18] Gorgoni, P. 2020. Suicide aid between life, self-determination, integrity and dignity of the sick person. *Giurisprudenza Italiana* 2020 (5): 1053.

[CR19] Guido Stampanoni Bassi. 2017. https://www.giurisprudenzapenale.com/processi/processo-nei-confronti-di-marco-cappato-suicidio-assistito-di-dj-fabo/

[CR21] Italian Constitutional Court. 2019. Decision No 242 of 22 November 2019 Constitutional appellate decision on cross-appeal. *Official Gazette* No 48 of 27 November 2019;

[CR20] Italian National Bioethics Committee Bioethical Reflections on Assisted Suicide 2019 available at https://bioetica.governo.it/it/pareri/pareri-e-risposte/ Accessed 21 August 2024

[CR22] Italian Supreme Court - First Civil Section - Judgment Oct. 16, 2007, No. 21748 https://www.biodiritto.org/ocmultibinary/download/2445/23238/9/a8c03fe6f441a32ecb6b9cfba437915c.pdf/file/21748_2007_Cass.pdf.Accessed 21 August 2024.

[CR23] Jox, R.J., Horn, R.J., and Huxtable, R. 2013. European perspectives on ethics and law in end-of-life care. In *Handbook of Clinical Neurology [Internet],*ed. Elsevier. [cited 2025 Mar 24], 155–165. Available from: https://linkinghub.elsevier.com/retrieve/pii/B9780444535016000135. 10.1016/B978-0-444-53501-6.00013-5 24182375

[CR26] Luchetti, M. 2010. Eluana Englaro, chronicle of a death foretold: Ethical considerations on the recent right-to-die case in Italy. *Journal of Medical Ethics* 36 (6): 333–5. 20439332 10.1136/jme.2009.034835

[CR27] Marrone, M., P. Berardi, B. Solarino, D. Ferorelli, S. Corradi, M. Silvestre, et al. 2022. Italian Legal Euthanasia: Unconstitutionality of the Referendum and Analysis of the “Italian” Problem. *Front Sociol* 12 (7): 898783. 10.3389/fsoc.2022.898783 PMC9315237 35903266

[CR28] Masoni R 2021. Cappato case and subsequent developments: an idem sentiment of ideas being affirmed? in Family and Personal Law, n 4

[CR29] Montanari, Vergallo G. 2019. The marco Cappato and Fabiano Antoniani (dj Fabo) case paves the way for new assisted suicide legislation in Italy: An overview of statutes from several European countries. *European Journal of Health Law* 26 (3): 221–39. 31220807 10.1163/15718093-12261428

[CR30] Moratti, S. 2010. The Englaro case: Withdrawal of treatment from a patient in a permanent vegetative state in Italy. *Cambridge Quarterly of Healthcare Ethics* 19 (3): 372–80. 20507685 10.1017/S0963180110000150

[CR31] Organic Law 3/2021, of 24 March, on the regulation of euthanasia. BOE no. 72, of March 25, 2021. 34037-49.

[CR32] Palermo, Fabris E. 2020. Il caso Cappato e l’aiuto medico a morire: profili penali. Nuova giur. civ. comm., 2020, 2, 431 ss.

[CR34] Solarino, B., F. Bruno, G. Frati, A. Dell’Erba, and P. Frati. 2011. A national survey of Italian physicians’ attitudes towards end-of-life decisions following the death of Eluana Englaro. *Intensive Care Medicine* 37 (3): 542–9. 21287145 10.1007/s00134-011-2132-5

[CR35] Sprung, C.L., M.A. Somerville, L. Radbruch, N.S. Collet, G. Duttge, J.P. Piva, et al. 2018. Physician-assisted suicide and euthanasia: Emerging issues from a global perspective. *Journal of Palliative Care* 33 (4): 197–203. 29852810 10.1177/0825859718777325

[CR36] Svenaeus, F. 2020. To die well: The phenomenology of suffering and end of life ethics. *Medicine, Health Care and Philosophy* 23 (3): 335–42. 31463881 10.1007/s11019-019-09914-6 PMC7426301

[CR40] Tripodina, C. 2008. Who gets to make key policy decisions on ethically controversial issues? (reflections on the sidelines of the “Englaro case”). *Giur. cost. fasc.* 5 (2008): 4069.

[CR41] Zagrebelsky, G. 2010. *Exchanging the Robe*. Laterza, Rome-Bari: State and Church in the government of man.

